# Association Between Na, K, and Lipid Intake in Each Meal and Blood Pressure

**DOI:** 10.3389/fnut.2022.853118

**Published:** 2022-03-04

**Authors:** Momoko Imamura, Hiroyuki Sasaki, Takae Shinto, Yu Tahara, Saneyuki Makino, Mai Kuwahara, Ayako Tada, Nanako Abe, Mikiko Michie, Shigenobu Shibata

**Affiliations:** ^1^Laboratory of Physiology and Pharmacology, School of Advanced Science and Engineering, Waseda University, Tokyo, Japan; ^2^Asken Inc., Tokyo, Japan

**Keywords:** blood pressure, dietary pattern, sodium, potassium, lipid, chrono-nutrition

## Abstract

Cardiovascular diseases (CVDs) are one of the leading causes of death worldwide, and one of the most significant risk factors for CVDs is high blood pressure. Blood pressure is associated with various nutrients, such as sodium, potassium, and cholesterol. However, research focusing on the timing of intake of these nutrients and blood pressure has not been conducted. In this study, we used dietary data and a questionnaire asking about the sleep, physical activity, and blood pressure, collected from the food-log app “Asken” (total *N* = 2,402), to investigate the relationship between the dietary data of nutrient intake in the breakfast, lunch, and dinner and blood pressure. Daily total intake of various nutrients such as sodium, sodium-to-potassium ratio, total energy, lipid, carbohydrate, and saturated fat showed a significant association with blood pressure depending on the meal timing. From multiple regression analysis, eliminating the confounding factors, lunch sodium-to-potassium ratio, dinner energy, lipid, cholesterol, saturated fat, and alcohol intake were positively associated with blood pressure, whereas breakfast protein and lunch fiber intake showed a negative association with blood pressure. Our results suggest that nutrient intake timing is also an important factor in the prevention of high blood pressure. Our study provides possibilities to prevent hypertension by changing the timing of nutrient intake, especially sodium, together with potassium and lipids. However, because our research was limited to food-log app users, broader research regarding the general population needs to be conducted.

## Introduction

Cardiovascular diseases (CVDs) are one of the leading causes of death worldwide, and one of the most significant risk factors for CVD is high blood pressure (1, 2). Blood pressure exhibits a circadian rhythm, rising from morning to afternoon and dipping at night (3). Mammals are under the control of this circadian rhythm, a rhythm of ~24 h, and the circadian clock mechanism plays an important role in physiological functions such as sleep/wakefulness, hormone secretion, and metabolism (4–6). In organs such as the kidney, peripheral clocks generate physiological rhythms (7). The circadian rhythm of blood pressure is driven by a complex molecular network of clock genes, and alterations in blood pressure rhythm from genetic manipulation of various clock genes have been reported in rodent studies (8). Mice lacking one of the core clock genes, Cryptochrome-1 and Cryptochrome-2, show salt-sensitive hypertension due to abnormally high synthesis of the mineral corticoid aldosterone, indicating a potential link between disturbances in the circadian rhythm and hypertension (9). Individuals with disrupted clocks, such as shift workers, showed higher blood pressure and a higher prevalence of CVDs (10). In patients with hypertension, loss of the day-night rhythm of blood pressure has been reported (11).

The blood pressure is well-controlled by urine and sodium excretion through the Na/K reuptake mechanism located in the renal tubule (12). Excess sodium intake and insufficient potassium intake have been shown to result in high blood pressure (13, 14). To observe the joint effects of sodium intake and potassium intake, the use of the sodium-to-potassium ratio (Na/K ratio) has been proposed in various studies. The Na/K ratio has been reported to show a stronger association with blood pressure than with sodium or potassium alone (15). Other nutrients have also been reported to be associated with hypertension. High cholesterol levels are known to cause arterial stiffness and arteriosclerosis, and systolic pressure is influenced by arterial stiffness (16). In addition, the benefits of dietary fiber intake on blood pressure have been reported (17). Reducing alcohol consumption lowers the blood pressure in a dose-dependent manner (18). Therefore, it is important to focus on various nutrients to prevent high blood pressure.

From transcriptome and metabolome analyses of human blood and tissue samples, it has been reported that circadian rhythms also exist in food digestion, absorption, and metabolism (8, 19). “Chrono-nutrition” is the study focusing on the intake timing of nutrients. There have been reports showing that nutrients show more health benefits depending on the intake timing (20). Catechin suppressed the elevation of postprandial glucose more effectively when taken in the evening than in the morning (21). Continuous beginning of the active phase administration of sesamin and episesamin improved lipid metabolism compared to administration at the end of the active phase (22). Morning intake of fish oil, which is abundant in docosahexaenoic acid (DHA) and eicosapentaenoic acid (EPA), suppressed serum triglyceride levels and decreased serum total saturated fatty acids and serum n-6 polyunsaturated fatty acids (23). Thus, it is important to consider not only what to eat but also when.

As previously stated, the association between blood pressure and the circadian clock has been suggested in various studies. Furthermore, the metabolism and excretion of nutrients associated with blood pressure exhibit a circadian rhythm. The excretion of sodium and potassium by the kidneys is controlled by the circadian timing system (24). Cholesterol metabolism also exhibits a 24-h rhythm (25). The influence of eating patterns and meal timings on blood pressure has also been suggested. Consuming meals irregularly has been suggested to be adversely associated with cardiometabolic risk, including blood pressure (26). Later lunch compared to the conventional Australian mealtime pattern showed higher blood pressure in women (27). Compared to breakfast eaters, elevated blood pressure was observed among female chronic breakfast skipping groups (28). Reversed feeding completely reversed the blood pressure rhythm in mice (29). These finding suggest that considering the prevention of high blood pressure from a “chrono-nutrition” point of view may be important.

According to the National Nutrition Survey in Japan (NNSJ) in 2019, compared to the estimated average requirement, the average intake of sodium and saturated fat is excessive, while that of potassium and dietary fiber is insufficient (30). Proposing the intake timing would be an effective way to approach the discrepancy between the actual and recommended intake of these nutrients. However, research focusing on blood pressure and the timing of intake of various nutrients has not been conducted. In this study, we aimed to investigate the relationship between dietary data of nutrient intake in three meals and blood pressure among ~2,400 users of “Asken,” a mobile health application for dietary management.

## Materials and Methods

### Study Participants and Mobile Health App “Asken”

Dietary data and questionnaire answers were collected through a popular food log and food coaching app, “Asken.” Based on the Dietary Intake Standards for Japanese determined by the Ministry of Health, Labor and Welfare, the app provides feedback on the dietary content of the meal, showing the excess and deficiency of nutrients.

The validity of Asken has been confirmed in previous studies. When comparing weighed dietary records with records from Asken, the energy and nutrient intakes were correlated, suggesting the validity of Asken (31). Paper-based dietary records and Asken records have been reported to have a 0.80 median correlation coefficient for nutrient intake (32). Those using this app are health-conscious people, and almost 95% of the users of the app aim to lose weight. This may account for 70% of the users being female. One limitation of using this app is self-efficacy. Dietary self-monitoring induces behavioral changes, and compared to paper-based records, electronic records have been reported to induce stronger changes (33, 34). Therefore, compared to those living under free conditions, our research was conducted on health-conscious people. The average daily nutritional data showed similarity with NNSJ, which reports the average daily Japanese nutritional intake (Supplementary Figure 1). We can obtain the NNSJ data only as the average. Lower cholesterol and carbohydrate intake and higher potassium, protein, and dietary fiber intake were observed, which may indicate the “health-conscious” characteristics of the participants compared to the average Japanese people.

In addition to these dietary records, an online survey was conducted in January 2021. This experiment was approved by the Ethics Review Committee on Research with Human Subjects at Waseda University (No. 2020-046) and followed the guidelines laid down in the Declaration of Helsinki. A total of 2491 participants responded at first, and by excluding data missing the reports of all three meals a day or missing basic characteristics such as body mass index (BMI) and sleep, physical activities, and subjects taking medicine, the final data were 707 for males and 1,695 for females.

### Questionnaire

From an online survey, the basic characteristics of the participants (age, gender, BMI) and other lifestyle-related factors (sleep, physical activity, blood pressure) were obtained.

### Assessment of Morning Type or Evening Type

Sleep factors such as morningness was assessed by MSFsc (sleep—corrected midpoint of sleep on free days). The computation was as follows: MSFsc = MSF—[(sleep duration on free days)—(sleep duration in workdays)]/2. This assessment is widely used and established (35).

### Physical Activity

From an online survey, physical activity was determined by the number of days and hours spent on the three types of activities (vigorous-intensity activity, moderate-intensity activity, and walking). We calculated weekly metabolic equivalents (MET) based on the International Physical Activity Questionnaire (IPAQ) analysis guidelines for each activity intensity for total physical activity (36). The IPAQ is widely used to assess physical activity (37).

### Blood Pressure

Participants answered the question; “Please answer the most recent record of maximum blood pressure (systolic blood pressure) by upper arm sphygmomanometer through medical checkup and/or at home.” Participants answered their systolic blood pressure by a score of 1–6 (1: <110 mmHg, 2: 111–120 mmHg, 3:121–130 mmHg, 4: 131–140 mmHg, 5: 141–150 mmHg, 6: over 151 mmHg). Blood pressure is usually recorded in the morning. We also asked if they were taking medication for high blood pressure, and the answers of those who answered yes were excluded.

### Dietary Data

The dietary data were analyzed using ~1-month average dietary records of the application. Energy content (kcal), protein, fat, carbohydrate, sodium, potassium, cholesterol, dietary fiber, saturated fat, and alcohol intake were measured for each of the three meals and snack. The intake timings of snacks might be different among participants, because of no check of snack time. From previous research, the validity of the dietary record of this app was high (31). The Na/K ratio was calculated by dividing the amount of sodium intake by potassium intake.

### Statistical Analysis

Statistical analysis of the obtained data was performed using a predictive analytics software for Windows (Statistical Package for the Social Sciences; IBM Corp., Chicago, IL, USA), and a *p*-value of <0.05 was considered statistically significant. To investigate the relationship between dietary patterns and blood pressure, Spearman's rank correlation analysis was conducted. To clarify the relationship excluding other factors, multiple regression analysis among the three meals for each nutrient was conducted. Data are expressed as mean and standard error.

## Results

### Subject Characteristics

**Figure 1:**
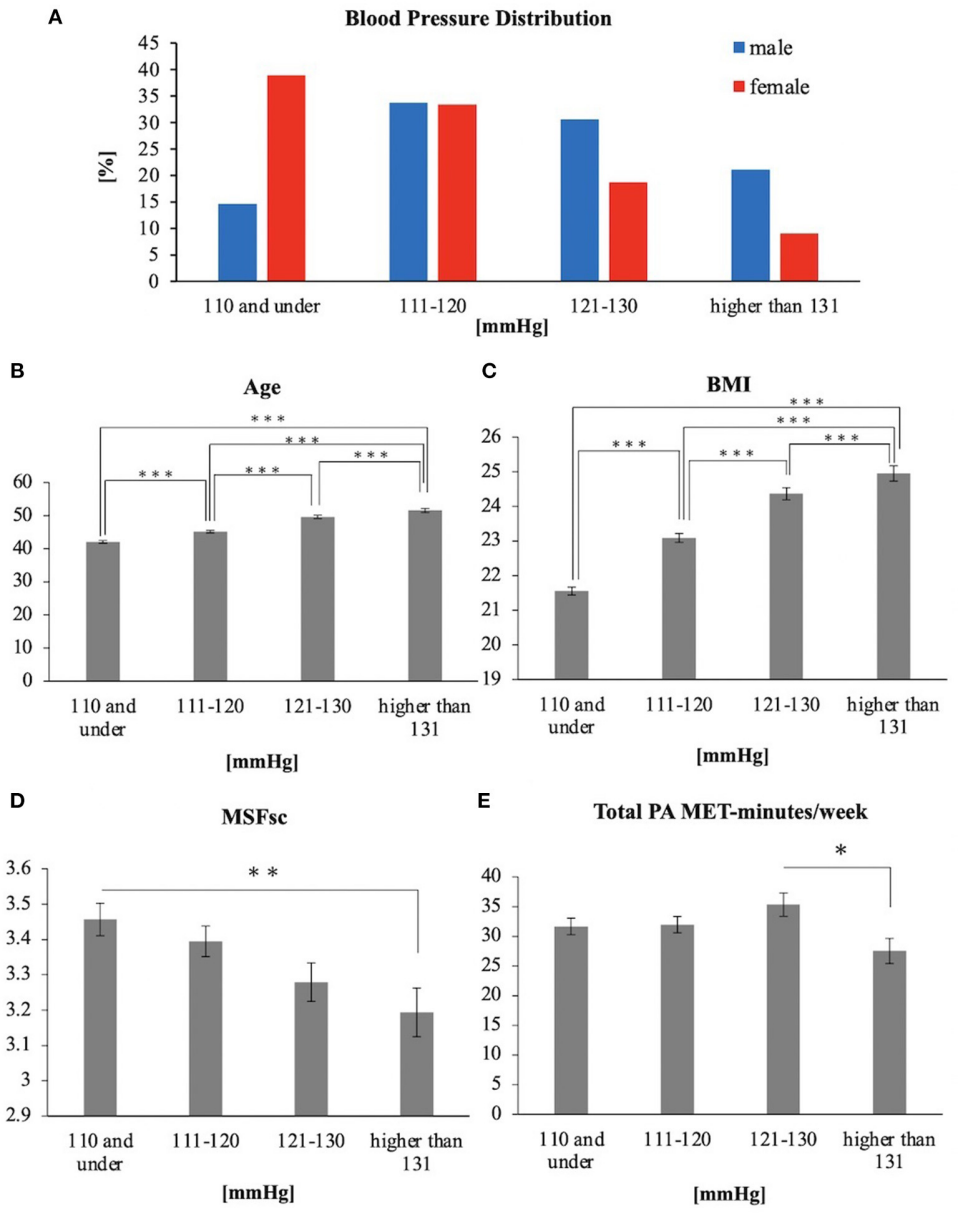
**(A)** Blood Pressure Distribution **(B)** Age **(C)** BMI **(D)** MSFsc **(E)** Total PA MET-minutes/week. *p < 0.05; **p < 0.01; ***p < 0.001 (Tukey).

For our analysis, we chose subjects from 2,491 responses to those who answered the questions regarding their blood pressure. The subjects were then excluded for various reasons, such as intake of medicine and outliers or unfilled data to 2,402 people. The mean (SE) age was 45.95 (0.237) years, BMI was 23.12 (0.0778), total PA MET min/week was 32.05 (0.818), and MSFsc was 3.36 (0.0254) (Table 1A). All values except MSFsc were significantly higher in men than in women. Large values of MSFsc in females demonstrated more eveningness than males. There were positive relationships between age, BMI, and blood pressure, and a negative relationship between MSFsc and blood pressure (Table 1B). Pearson's chi-square test revealed that blood pressure distribution was different between males and females (*p* < 0.001), and males exhibited higher blood pressure than females (Figure 1A). There was a positive relationship between age and blood pressure (Figure 1B) and between BMI and blood pressure (Figure 1C). However, there was a negative relationship between MSFsc and blood pressure (Figure 1D). Although there was no relationship between physical activity and blood pressure, individuals with the highest blood pressure showed low physical activity (Figure 1E).

**Table 1:** Basic characteristics of participants and correlation analysis of basic characteristics and blood pressure.

**(A) Basic characteristic**							
	**All (*****N*** **= 2,402)**		**Male (*****N*** **= 707)**		**Female (*****N*** **= 1,695)**		***P*** **-value**
	**Mean**	**SE**	**Mean**	**SE**	**Mean**	**SE**	**(Male vs. female)**
Age	45.95	0.237	50.14	0.406	44.17	0.279	<0.001
BMI (kg/m^2^)	23.12	0.0778	24.21	0.129	22.66	0.094	<0.001
Total PA MET-minutes/week	32.05	0.818	37.55	1.601	29.64	0.939	<0.001
MSFsc	3.363	0.0254	3.076	0.0453	3.489	0.0302	<0.001
**(B) Correlation analyses of basic characteristics and blood pressure**							
	**Male (*****N*** **= 707)**		**Female (*****N*** **= 1,695)**				
	**Correlation**	***P*** **-value**	**Correlation**	***P*** **-value**			
Age	0.213^**^	<0.001	0.264^**^	<0.001			
BMI(kg/m^2^)	0.226^**^	<0.001	0.315^**^	<0.001			
Total PA MET-minutes/week	−0.100^**^	0.007	−0.03	0.212			
MSFsc	−0.008	0.839	−0.033	0.166			

** *p < 0.01 by Spearman's rank correlation coefficient. BMI, Body Mass Index; PA, Physical Activity; MET, Metabolic Equivalents; MSFsc, sleep-corrected Midpoint of sleep in free-days*.

### Nutrient Intake Volumes in Each Meal

Next, the intake volumes of each nutrient were compared among breakfast, lunch, and dinner in male and female participants (Supplementary Tables 1, 2). Intake volumes of snack are shown in Supplementary Table 3. All nutrients except for carbohydrates and alcohol in females were significantly taken at dinner and next at lunch in comparison with breakfast in both males and females.

### Correlations Analyses of Intake of Nutrients and Blood Pressure

Correlation analysis of the intake of various nutrients and blood pressure was applied for each meal (breakfast, lunch, and dinner) and snack. In addition to the amount of intake for each nutrient, the mean energy ratios of protein, fat, and carbohydrate were compared for each meal and snack. Spearman's correlation analysis showed that blood pressure had a strong positive association with the Na/K ratio for every meal in both males and females (Table 2). Nutrients that showed positive associations with blood pressure in both males and females were lunch sodium, lunch and dinner energy, total lipid, lunch carbohydrate, and total and dinner alcohol. Lunch and snack protein ratio and total, dinner and snack dietary fiber intake were negatively associated with blood pressure in both sexes. In females, lipid intake showed a dinner-specific positive correlation (Table 2).

**Table 2:** Correlations analyses of intake of nutrients and blood pressure.

	**Male**					**Female**				
	**Total**	**Breakfast**	**Lunch**	**Dinner**	**Snack**	**Total**	**Breakfast**	**Lunch**	**Dinner**	**Snack**
Na/K	0.128^**^	0.090^*^	0.117^**^	0.128^**^	0.039	0.051^*^	0.053^*^	0.105^**^	0.051^*^	0.009
Sodium	0.087^*^	0.044	0.097^**^	0.087^*^	−0.01	0.023	0.055^*^	0.065^**^	0.023	−0.022
Potassium	−0.06	−0.044	−0.062	−0.06	−0.080^*^	−0.02	−0.023	−0.036	−0.02	−0.029
Energy	0.065	−0.011	0.074^*^	0.106^**^	−0.019	0.106^**^	0.052^*^	0.070^**^	0.079^**^	−0.018
Protein	−0.037	−0.062	−0.057	0.012	−0.07	−0.003	−0.04	−0.026	0.021	−0.053^*^
Lipid	0.085^*^	0.034	0.055	0.059	−0.029	0.096^**^	0.043	0.046	0.082^**^	−0.041
Carbohydrate	0.03	0.005	0.084^*^	−0.01	−0.007	0.074^**^	0.068^**^	0.067^**^	0.041	0.013
Protein (PFC ratio)	−0.058	−0.049	−0.110^**^	0.019	−0.081^*^	−0.079^**^	−0.094^**^	−0.077^**^	−0.04	−0.055^*^
Fat (PFC ratio)	0.061	0.066	0.019	0.067	0.006	0.045	0.013	0.024	0.054^*^	−0.041
Carbohydrate (PFC ratio)	0.003	−0.019	0.068	−0.041	0.052	0.036	0.064^**^	0.048^*^	0.006	0.063^**^
Cholesterol	−0.025	−0.01	−0.067	0.05	−0.016	0.024	0.01	−0.002	0.028	0.028
Dietary Fiber	−0.086^*^	−0.055	−0.063	−0.083^*^	−0.075	−0.066^**^	−0.057^*^	−0.060^*^	−0.051^*^	−0.049^*^
Saturated Fat	0.054	0.044	0.00	0.067	−0.024	0.097^**^	0.061^*^	0.037	0.096^**^	−0.02
Alcohol	0.077^*^	0.035	0.038	0.113^**^	0.058	0.063^**^	0.006	0.052^*^	0.065^**^	−0.003

* *p < 0.05;*

** *p < 0.01 by Spearman's rank correlation coefficient*.

### Association Between the Timing of Each Nutrient and Blood Pressure

Multiple regression analysis showed that even when eliminating the effects of confounding factors, such as sex, age, BMI, MSFsc, and PA MET min/week, various nutrients showed different associations with blood pressure depending on their intake timing (Table 3). Focusing on salt intake, lunch and snack Na/K ratio showed a significant positive association, and lunch potassium intake showed a negative association (Table 3). Dinner energy, dinner lipid, dinner cholesterol, dinner saturated fat, and dinner alcohol intake were positively associated with blood pressure, whereas breakfast protein and lunch fiber intake showed a negative association with blood pressure (Table 4).

**Table 3:** Association between the timing of salt intake and blood pressure including snack.

	**β**	***P*-value**	***R*2**	***F***
Breakfast_Na/K	−0.001	0.958	0.187	53.495
Lunch_Na/K	0.072	0.001		
Dinner_Na/K	−0.014	0.514		
Snack_Na/K	0.046	0.022		
Breakfast_Sodium	0.0048	0.812	0.177	52.405
Lunch_Sodium	0.034	0.110		
Dinner_Sodium	0.014	0.518		
Snack_Na/K	−0.018	0.371		
Breakfast_Potassium	−0.017	0.414	0.185	53.764
Lunch_Potassium	−0.043	0.037		
Dinner_Potassium	0.000	0.990		
Snack_Potassium	−0.029	0.142		

*Multivariable regression analyses adjusted by age, sex, BMI, Total PA MET-minutes/week and MSFsc*.

**Table 4:** Association between the timing of nutrient intake and blood pressure including snack.

	**β**	***P*-value**	***R*2**	***F***
Breakfast_Energy	0.007	0.735	0.183	53.700
Lunch_Energy	0.020	0.366		
Dinner_Energy	0.100	<0.0001		
Snack_Energy	0.008	0.703		
Breakfast_Protein	−0.046	0.027	0.183	53.412
Lunch_Protein	−0.032	0.137		
Dinner_Protein	0.019	0.393		
Snack_Protein	−0.017	0.405		
Breakfast_Lipid	0.005	0.792	0.185	54.885
Lunch_Lipid	0.016	0.449		
Dinner_Lipid	0.059	0.007		
Snack_Lipid	−0.013	0.515		
Breakfast_Carbohydrate	0.018	0.382	0.183	53.874
Lunch_Carbohydrate	0.034	0.125		
Dinner_Carbohydrate	0.009	0.699		
Snack_Carbohydrate	0.007	0.725		
Breakfast_Cholesterol	−0.028	0.165	0.179	52.691
Lunch_Cholesterol	−0.025	0.215		
Dinner_Cholesterol	0.041	0.049		
Snack_Cholesterol	−0.001	0.960		
Breakfast_Fiber	−0.034	0.108	0.184	53.605
Lunch_Fiber	−0.048	0.026		
Dinner_Fiber	−0.023	0.285		
Snack_Fiber	−0.026	0.192		
Breakfast_Saturated Fat	0.013	0.523	0.186	55.343
Lunch_Saturated Fat	−0.005	0.814		
Dinner_Saturated Fat	0.072	0.001		
Snack_Saturated Fat	−0.008	0.676		
Breakfast_Alcohol	−0.013	0.509	0.179	51.548
Lunch_Alcohol	0.022	0.304		
Dinner_Alcohol	0.041	0.065		
Snack_Alcohol	0.015	0.475		

*Multivariable regression analyses adjusted by age, sex, BMI, Total PA MET-minutes/week and MSFsc*.

## Discussion

In this study, we investigated the association between blood pressure and different nutrient intake timing among mobile health app “Asken” male and female users. Our findings confirm previous findings that high Na/K ratio, high lipid intake, high alcohol intake, and low dietary fiber are associated with high blood pressure (15, 17, 18). However, little is known about the influence of intake timing. Our research provides new perspectives on the prevention of hypertension and has shown that various nutrients are associated with blood pressure at different meal timings. In both males and females, lunch and snack Na/K ratio, dinner energy, dinner lipid, and dinner saturated fatty acids showed a meal-specific positive association with blood pressure, and breakfast protein, lunch potassium and lunch dietary fiber showed a meal-specific negative association with blood pressure after eliminating the confounding factors.

From previous studies, dietary records collected by Asken have been reported to be an effective method for estimating the energy and nutrient intakes of Japanese women (31). As stated, the nutrition amount resembles the data calculated from the NNSJ (30) (Supplementary Figure 1 and Shinto et al.). Focusing on sodium and potassium, dinner sodium intake was lower and potassium intake was higher, resulting in a lower dinner Na/K ratio than that of NNSJ (Supplementary Figure 2). This can be explained by the characteristics of the Asken users. With the aim of losing weight, Asken users have higher health consciousness and high self-efficacy (38).

Our results showed that blood pressure had a strong positive association with the Na/K ratio, especially the lunch and snack Na/K ratio. In addition, the negative correlation of lunch potassium shows that promoting excretion of sodium is important for lowering blood pressure at lunch time. Sodium and potassium urine excretion has been previously reported to exhibit circadian rhythm, which is explained by the rhythm of aldosterone (24). Aldosterone, a mineralocorticoid that through ENaC is responsible for the reabsorption of Na and the increase in K secretion through K channels in the distal nephron, has also been reported to exhibit rhythm, low at nighttime and high in the morning (39). Therefore, intake of sodium at dinner time may be excluded in urine, even though sodium intake and the Na/K ratio were high in the dinner. Several previous studies have demonstrated higher excretion of sodium and an increase of Na/K ratio in the evening than in the morning and afternoon (24).

Potassium and dietary fiber are both rich in vegetables. Lunch fiber and potassium intake showed a negative correlation with blood pressure in the current experiment, suggesting the intake of more vegetables at lunch and snack time to protect higher blood pressure. Higher blood pressure has been reported to be associated with lower gut microbiota alpha diversity in many human cross-sectional studies (40). A low intake of dietary fiber leads to reduced microbial diversity (41). Inulin, a water-soluble dietary fiber, has been reported to have greater positive effects on the microbiota in the morning than in the evening (42). Taken together, these results suggest that dietary fibers, especially at lunch and breakfast time, may be more helpful than at dinner time to protect higher blood pressure through an increase in microbial diversity. These results have suggested the importance of vegetables in the prevention of hypertension in a time-specific manner. Vegetables are also known to include many dietary polyphenols such as flavonoids, and their treatment or prevention of hypertension (43). As previously stated, catechin, a type of flavonoid, suppressed the elevation of postprandial glucose more effectively when taken in the evening than in the morning, suggesting that the metabolic response of polyphenols may depend on their intake timing (21). Dietary polyphenols' health promoting effects are reported to be in a hormetic dose-response manner, partly via the upregulation of Nrf2 pathway (44–47). Therefore, in addition to the analysis of nutrients that we have conducted, analysis of the intake timing and volume of dietary polyphenols may provide a clearer view.

Furthermore, breakfast protein was negatively correlated with blood pressure. The beneficial effect of proteins on blood pressure is small, according to previous studies (48). However, a high-protein breakfast has been suggested to accelerate overloading-induced skeletal muscle hypertrophy in mice and have greater skeletal muscle volume in human studies (49). It has also been revealed that breakfast and lunch proteins have a strong positive association with daily physical activity (unpublished observation), and recent studies have supported the role of physical activity in the prevention of hypertension (50). Therefore, the relationship between morning protein intake and exercise may explain its negative correlation with blood pressure. As clinical trials of food-protein-derived peptides in the management of hypertension have been published (51), we should examine the detailed information of protein compositions (cereals, beans, vegetables, fruits, meat, fish milk, egg, etc.) in future experiment.

Dinner cholesterol and saturated fatty acids were positively correlated with blood pressure. A high dinner energy also showed a positive correlation. It is well-known that a high intake of cholesterol and saturated fatty acids causes progression of arteriosclerosis and higher blood pressure (52, 53). However, we do not know the true reason why dinner time intake of these nutrients and energy are related to high blood pressure. As cholesterol synthesis in the body is high in the evening, cholesterol intake and synthesized cholesterol may cooperatively promote arteriosclerosis progression. The intake of saturated fatty acids and energy at dinner time cannot be consumed as an energy source because of sleep. Excess circulation of triglycerides and fatty acids may accelerate the progression of arteriosclerosis (53).

High energy intake and lipid intake at dinner are reported to lead to obesity, which is known to be strongly associated with blood pressure (16). In the present study, we found a positive relationship between BMI and blood pressure. However, the correlation of energy/lipid at dinner time was still observed when eliminating the obesity factor in the present experiment. It has been reported that early time-restricted feeding, with dinner before 3 pm, led to lower blood pressure without inducing weight loss (54). This study, along with our present data, strongly suggests that taking energy/lipid-rich dinner at an earlier clock time may prevent higher blood pressure. As it is well-known that hypertension is related with seriousness of diabetes and dyslipidemia, we should investigate the relationships between intake of Na, K lipid, and saturated fatty acids and these diseases in future. In the current experiment, we have not asked if participants had these diseases or not.

There are some limitations to our study. First, the dietary data were collected through self-reports by the subjects, and this self-reporting may have resulted in self-efficacy. Previous studies have discussed that self-monitoring results in behavioral changes (34). The second is blood pressure measurement. Blood pressure was reported by the participants themselves, and this may cause some inaccuracy in the data. We also could not measure the blood pressure at the same time. For more precise data, blood pressure needs to be measured at a standardized time, such as in the morning 1 h after awakening and in the evening before going to sleep according to the Japanese Society of Hypertension have presented in the guidelines for home blood pressure measurement (55). Third, only systolic blood pressure has been reported because many Japanese are unconcerned about their diastolic blood pressure. As both systolic and diastolic blood pressure are used for hypertension diagnosis, in future research, both systolic and diastolic blood pressure should be investigated. Fourth, we could not check the intake timing of snack, however similarity to lunch Na/K ratio suggests many participants may take snack at afternoon. Fifth, the characteristics of the participants being highly health-conscious may have affected the results. Lastly, we could only investigate saturated fatty acids this time, but by looking at the amount of DHA and EPA that work in suppressing arteriosclerosis, we would have been able to have a better understanding.

## Conclusions

We analyzed the relationship between the intake timing of nutrients and blood pressure. In both males and females, a positive association between meals and blood pressure was observed in the Na/K ratio (lunch), energy (dinner), lipid (dinner), and saturated fatty acids (dinner). Moreover, a meal-specific negative association with blood pressure was seen in protein (breakfast), potassium (lunch), and dietary fiber (lunch). Our study provides possibilities to prevent hypertension by changing the timing of various nutrient intakes, especially sodium together with potassium and lipids. However, an intervention study to investigate the effect of the timing of each nutrient on blood pressure needs to be conducted in the future.

## Data Availability Statement

The raw data supporting the conclusions of this article will be made available by the authors, without undue reservation.

## Ethics Statement

This experiment was approved by the Ethics Review Committee on Research with Human Subjects at Waseda University (No. 2020-046) and followed the guidelines laid down in the Declaration of Helsinki. The patients/participants provided their written informed consent to participate in this study.

## Author Contributions

MI, HS, YT, TS, SM, MK, AT, NA, MM, and SS designed the research and analyzed the data. MI and SS wrote the manuscript. All authors contributed to the article and approved the submitted version.

## Funding

This research was funded by the Japan Society for the Promotion of Science (JSPS) KAKENHI (Kiban A) and the JST-Mirai Program (Grant Number: JMPJM120D5) by SS.

## Conflict of Interest

MM is a corporate officer in Asken Inc. AT and NA are employees of Asken Inc. The remaining authors declare that the research was conducted in the absence of any commercial or financial relationships that could be construed as a potential conflict of interest.

## Publisher's Note

All claims expressed in this article are solely those of the authors and do not necessarily represent those of their affiliated organizations, or those of the publisher, the editors and the reviewers. Any product that may be evaluated in this article, or claim that may be made by its manufacturer, is not guaranteed or endorsed by the publisher.
