# Supplementary material for: Association Between Na, K, and Lipid Intake in Each Meal and Blood Pressure
Source: Front Nutr. 2022 Mar 4;9:853118. doi: 10.3389/fnut.2022.853118 (PMC8931534; doi:10.3389/fnut.2022.853118)
Supplement: Supplementary file 1 [file Data_Sheet_1.docx]

Supplementary Material

# Supplementary Material

## Supplementary Figures

**Supplemental Fig.1 Comparison of nutritional intake between Asken and National Nutrition Survey in Japan (NNSJ)** (A)Daily Energy (B) Daily Sodium intake (C) Daily Potassium intake (D) Daily Protein intake (E)Daily Fat intake (F) Daily Carbohydrates intake (G) Daily Cholesterol intake (H)Dietary Fiber intake (I)Daily Saturated Fatty Acids intake

**Supplemental Fig.2 Comparison of Sodium and Potassium intake in each meal between Asken and National Nutrition Survey in Japan (NNSJ)** (A)Sodium Intake (Male) (B) Potassium Intake (Male) (C) Na/K ratio (Male) (D) Sodium Intake (Female) (E) Potassium Intake (Female) (F) Na/K ratio (Female)

## Supplementary Table

Supplemental Table 1. Characteristics of participants (Energy and three macronutrients)

|  |  |  |  |  |  |  |  |  |  | |  | |  | |  | |
| --- | --- | --- | --- | --- | --- | --- | --- | --- | --- | --- | --- | --- | --- | --- | --- | --- |
|  |  | All (N=2402) | | |  | Male (N=707) | | |  | | Female (N=1695) | | | | | |
|  |  | Mean | SE | Significance |  | Mean | SE | Significance |  | | Mean | | SE | | Significance | |
| Total_energy (kcal/d) | | 1527.5 | 7.952 |  |  | 1752.1 | 17.97 |  |  | 1429.2 | | 7.114 | |  | |  |
| Breakfast_energy (kcal) | | 380.31 | 2.881 | ### |  | 435.8 | 5.700 | ### |  | 357.3 | | 3.158 | | ### | |  |
| Lunch_energy (kcal) | | 556.17 | 2.846 | *** |  | 633.1 | 5.395 | *** |  | 524.10 | | 3.027 | | *** | |  |
| Dinner_energy (kcal) | | 636.10 | 4.311 | *** ### |  | 808.4 | 7.905 | *** ### |  | 565.56 | | 4.055 | | *** ### | |  |
| Protein intake (g/d) | | 65.85 | 0.365 |  |  | 73.26 | 0.812 |  |  | 62.61 | | 0.361 | |  | |  |
| Breakfast_Protein (g) | | 16.35 | 0.155 | ### |  | 18.57 | 0.310 | ### |  | 15.44 | | 0.174 | | ### | |  |
| Lunch_Protein (g) | | 22.74 | 0.140 | *** |  | 25.19 | 0.284 | *** |  | 21.73 | | 0.153 | | *** | |  |
| Dinner_Protein (g) | | 28.70 | 0.185 | *** ### |  | 34.71 | 0.363 | *** ### |  | 26.21 | | 0.183 | | *** ### | |  |
| Fat intake (g/d) | | 52.73 | 0.307 |  |  | 59.68 | 0.660 |  |  | 49.69 | | 0.307 | |  | |  |
| Breakfast_Fat (g) | | 12.06 | 0.117 | ### |  | 13.81 | 0.239 | ### |  | 11.33 | | 0.129 | | ### | |  |
| Lunch_Fat (g) | | 18.901 | 0.127 | *** |  | 21.18 | 0.246 | *** |  | 17.95 | | 0.141 | | *** | |  |
| Dinner_Fat (g) | | 23.21 | 0.170 | *** ### |  | 28.48 | 0.315 | *** ### |  | 21.03 | | 0.176 | | *** ### | |  |
| Carbohydrate intake (g) | | 185.6 | 1.049 |  |  | 210.1 | 2.289 |  |  | 174.9 | | 1.028 | |  | |  |
| Breakfast_Carbohydrate (g) | | 51.92 | 0.420 | ### |  | 58.53 | 0.840 | ### |  | 49.18 | | 0.465 | | ### | |  |
| Lunch_Carbohydrate (g) | | 71.39 | 0.396 | *** |  | 81.57 | 0.771 | *** |  | 67.12 | | 0.418 | | *** | |  |
| Dinner_Carbohydrate (g) | | 67.25 | 0.496 | *** ### |  | 82.74 | 0.921 | *** |  | 60.78 | | 0.514 | | *** ### | |  |
| **p<0.01, ***p<0.001 (vs. Breakfast, Tukey) | | | | | | | | | | | | | | | |  |
| ###p<0.001 (vs. Lunch, Tukey) | | | | | | | | | | | | | | | |  |

Supplemental Table 2. Characteristics of participants (Other nutrients)

|  |  |  | | |  |  | | |  |  | | | |
| --- | --- | --- | --- | --- | --- | --- | --- | --- | --- | --- | --- | --- | --- |
|  |  | All (N=2402) | | |  | Male (N=707) | | |  | Female (N=1695) | | | |
|  |  | Mean | SE | Significance |  | Mean | SE | Significance |  | Mean | SE | Significance |  |
| Sodium intake (mg/d) | | 3241.9 | 19.46 |  |  | 3694.2 | 41.84 |  |  | 3044.0 | 19.33 |  |  |
| Breakfast_Sodium (mg) | | 620.7 | 7.524 | ### |  | 718.8 | 15.43 | ### |  | 580.2 | 8.311 | ### |  |
| Lunch_Sodium (mg) | | 1268.5 | 8.894 | *** |  | 1476.2 | 17.72 | *** |  | 1181.6 | 9.462 | *** |  |
| Dinner_Sodium (mg) | | 1431.0 | 10.04 | *** ### |  | 1700.5 | 18.39 | *** ### |  | 1317.2 | 10.88 | *** ### |  |
| Potassium intake (mg/d) | | 2232.8 | 13.37 |  |  | 2340.4 | 28.26 |  |  | 2185.7 | 14.58 |  |  |
| Breakfast_Potassium (mg) | | 625.2 | 6.563 | ### |  | 675.2 | 13.12 | ## |  | 604.8 | 7.465 | ### |  |
| Lunch_Potassium (mg) | | 712.4 | 4.957 | ** |  | 725.2 | 9.458 | ** |  | 707.0 | 5.811 | *** |  |
| Dinner_Potassium (mg) | | 968.1 | 6.411 | *** ### |  | 1093.4 | 12.20 | *** ### |  | 915.8 | 7.162 | *** ### |  |
| Cholesterol intake (g/d) | | 223.5 | 1.842 |  |  | 241.6 | 3.931 |  |  | 215.6 | 1.985 |  |  |
| Breakfast_Cholesterol (g) | | 53.2 | 0.988 | ### |  | 57.4 | 1.99 | ### |  | 51.49 | 1.125 | ### |  |
| Lunch_Cholesterol (g) | | 77.0 | 0.851 | *** |  | 80.5 | 1.68 | *** |  | 75.57 | 0.979 | *** |  |
| Dinner_Cholesterol (g) | | 99.5 | 0.933 | *** ### |  | 118.4 | 1.94 | *** ### |  | 91.58 | 0.983 | *** ### |  |
| Dietary fiber intake (g/d) | | 17.8 | 0.119 |  |  | 18.6 | 0.237 |  |  | 17.48 | 0.135 |  |  |
| Breakfast_Dietary fiber (g) | | 5.16 | 0.0664 | ### |  | 5.52 | 0.13 | ### |  | 5.003 | 0.0778 | ### |  |
| Lunch_Dietary fiber (g) | | 6.01 | 0.0429 | *** |  | 6.14 | 0.0789 | *** |  | 5.949 | 0.0511 | *** |  |
| Dinner_Dietary fiber (g) | | 7.24 | 0.0511 | *** ### |  | 8.06 | 0.0946 | *** ### |  | 6.90 | 0.0588 | *** ### |  |
| Saturated Fat intake (g/d) | | 14.0 | 0.0922 |  |  | 15.6 | 0.191 |  |  | 13.4 | 0.0987 |  |  |
| Breakfast_Saturated Fat (g) | | 3.63 | 0.0403 | ### |  | 4.04 | 0.0807 | ### |  | 3.47 | 0.0455 | ### |  |
| Lunch_Saturated Fat (g) | | 4.88 | 0.0373 | *** |  | 5.28 | 0.0702 | *** |  | 4.71 | 0.0434 | *** |  |
| Dinner_Saturated Fat (g) | | 5.90 | 0.0483 | *** ### |  | 7.21 | 0.0922 | *** ### |  | 5.36 | 0.0512 | *** ### |  |
| Alcohol intake (g/d) | | 4.95 | 0.166 |  |  | 7.36 | 0.377 |  |  | 3.89 | 0.166 |  |  |
| Breakfast_Alcohol (g) | | 0.115 | 0.0047 | ### |  | 0.144 | 0.0104 | ### |  | 0.103 | 0.0049 | ### |  |
| Lunch_Alcohol (g) | | 0.579 | 0.0174 | ** |  | 0.736 | 0.0374 |  |  | 0.514 | 0.019 | ** |  |
| Dinner_Alcohol (g) | | 4.49 | 0.166 | ** ### |  | 7.39 | 0.403 | *** ### |  | 3.35 | 0.160 | *** ### |  |
| **p<0.01, ***p<0.001 (vs. Breakfast, Tukey) | | | | | | | | | | | | |  |
| ###p<0.001 (vs. Lunch, Tukey) | | | | | | | | | | | | |  |

Supplemental Table 3. Snack nutritional intake of participants

|  |  | |  |  | |  |  | |
| --- | --- | --- | --- | --- | --- | --- | --- | --- |
|  | All (N=2402) | |  | Male (N=707) | |  | Female (N=1697) | |
|  | Mean | SE |  | Mean | SE |  | Mean | SE |
| Energy(kcal) | 234.1 | 2.44 |  | 259.1 | 5.32 |  | 224.2 | 2.63 |
| Protein(g) | 6.918 | 0.105 |  | 8.159 | 0.248 |  | 6.431 | 0.106 |
| Fat(g) | 9.244 | 0.116 |  | 9.803 | 0.246 |  | 9.017 | 0.128 |
| Carbohydrate(g) | 30.07 | 0.319 |  | 32.12 | 0.698 |  | 29.24 | 0.346 |
| Sodium(mg) | 172.6 | 2.73 |  | 209.3 | 6.24 |  | 157.9 | 2.82 |
| Potassium(mg) | 246.6 | 3.18 |  | 273.7 | 6.59 |  | 235.6 | 3.56 |
| Cholesterol(g) | 16.60 | 0.33 |  | 16.54 | 0.667 |  | 16.62 | 16.6 |
| Dietary Fiber(g) | 1.860 | 0.0293 |  | 1.968 | 0.0622 |  | 1.816 | 1.82 |
| Saturated Fat(g) | 2.964 | 0.0398 |  | 2.937 | 0.0798 |  | 2.974 | 2.97 |
| Alcohol(g) | 0.424 | 0.0356 |  | 0.853 | 0.0989 |  | 0.251 | 0.251 |
|  |  |  |  |  |  |  |  |  |
